# Shot Distribution and Accuracy in Senior and Youth International Basketball Games: Changes over the Decade of the 2010s

**DOI:** 10.3390/ijerph18189900

**Published:** 2021-09-20

**Authors:** Haruhiko Madarame

**Affiliations:** Department of Sports and Fitness, Shigakkan University, 55 Nakoyama, Yokonemachi, Obu 474-8651, Aichi, Japan; madarame-tky@umin.ac.jp

**Keywords:** basketball, game-related statistics, youth sports

## Abstract

This study aimed to investigate the changes in shot distribution and accuracy in senior and under-17 international basketball tournaments in the 2010s. A retrospective cross-sectional design was employed, and a total of 1055 games from 20 world-level tournaments held between 2010 and 2019 were analyzed. The tournaments held in 2010 were played under the old three-point line (6.25 m), and the rest of the tournaments were played under the new three-point line (6.75 m). The numbers of two- and three-point shot attempts in each game were normalized to 100 possessions. Differences in mean values of two- and three-point shot attempts were analyzed by a two-way (category × year) between-subjects analysis of variance (ANOVA). Differences in the success rates of two- and three-point shots were analyzed by Fisher’s exact test. There was no significant category × year interaction for two-point shot attempts. However, there were significant main effects of category and year for two-point shot attempts. The number of two-point shot attempts increased significantly in 2012 and, in 2016, returned to a level not significantly different from that in 2010, independent of categories. There was a significant category × year interaction for three-point shot attempts. The number of three-point shot attempts decreased significantly in 2012 and, by 2016, returned to a level not significantly different from that in 2010 in senior men and under-17 women. There was no significant change in the number of three-point shot attempts in under-17 men. The effect of the extension of the three-point line on shot accuracy was limited. Differences in shot accuracy were prominent between age categories: the accuracy was lower in under-17 games than in senior games for both sexes. Although under-17 men attempted fewer three-point shots than senior men, under-17 women attempted a similar number of or more three-point shots than senior women. These findings can contribute to a better understanding of age and sex differences in recent trends in international basketball.

## 1. Introduction

Since its invention in 1891 [1], basketball has undergone many changes throughout its history. Changes in the rules and the development of new tactics have changed the nature of the game. There is no doubt that the three-point shot is one of the essential elements of the modern basketball game. A shot made from outside the three-point line is worth 1.5 times as much as a shot made from inside the line; therefore, three-point shots have a significant impact on the game’s outcome. Changing the distance of the three-point line from the basket has been shown to alter the distribution of two- and three-point shot attempts in professional league games [2,3]. The National Basketball Association (NBA) adopted the three-point line in the 1979–1980 season: the distance from the center of the basket was 6.71 m (22 feet) at the corners and 7.24 m (23 feet 9 inches) at the top of the key. When the distance was shortened to a uniform 6.71 m in the 1994–1995 season, the number of three-point shot attempts increased, and two-point shot attempts decreased [2]. In contrast, when the three-point line returned to the original distance in the 1997–1998 season, the number of three-point shot attempts decreased, and two-point shot attempts increased [2]. A similar change was observed in the EuroLeague, arguably the second-highest-ranked professional league in the world behind the NBA. Since the EuroLeague has basically been played under the rules of the International Basketball Federation (FIBA), the distance of the three-point line was increased from 6.25 m to 6.75 m in the 2010–2011 season according to FIBA’s new rules. Štrumbelj et al. [3] reported a significant decrease in the number of three-point shot attempts and a significant increase in the number of two-point shot attempts after the rule change.

Rule changes are not the only cause of altering shot distribution. There has been a remarkable rise in the percentage of three-point shot attempts in the NBA since the mid-2010s. The percentage of three-point shot attempts throughout the NBA increased from 28.5% in the 2015–2016 season to 38.4% in the 2019–2020 season, and the Houston Rockets became the first team in NBA history to attempt more three-point shots than two-point shots in a single season in the 2017–2018 season. There were no changes to the rules regarding the three-point shot during that period. It is believed that the rise in the percentage of three-point shot attempts is due to the fact that more teams are focusing on efficiency based on sports analytics [4,5].

Elite players also compete in international tournaments such as the Olympics and the World Cup. Since international tournaments are held over a short period of time and there is limited time for national team players to practice together, the games may be played differently in international tournaments than in professional leagues. Another feature of international tournaments is that there are categories not only for senior players but for youth players. Clarifying the characteristics of elite youth games in comparison with senior games will contribute to the long-term development of elite players because players in youth national teams are expected to transition to senior national teams. Pérez-Ferreirós et al. [6] reported the effects of the 2010 rule changes on senior and youth European championships for men and women. The study is highly novel in that it used an interrupted time series analysis, probably for the first time, to assess the effects of rule changes in sports. However, the latest tournaments analyzed in the study were 2015 for seniors and 2016 for youths: the study did not include the late 2010s, when the percentage of three-point shot attempts skyrocketed in the NBA. Since the NBA is the highest level of basketball league globally, the NBA trend might spread to international tournaments. Another concern is that combining three age categories (under-16, under-18, under-20) as a youth category in the study might make it challenging to detect features of younger age players. Differences in game characteristics between under-17 and under-19 world championships have been reported [7]. Therefore, this study aimed to investigate the changes in shot distribution and accuracy in senior and under-17 international basketball tournaments in the 2010s.

## 2. Materials and Methods

### 2.1. Sample and Variables

This study analyzed 1055 games from 20 world-level tournaments held between 2010 and 2019 (Table 1). The tournaments held in 2010 were played under the old three-point line (6.25 m), and the rest of the tournaments were played under the new three-point line (6.75 m). The ball used in the men’s game (size 7) was slightly larger and heavier than the ball used in the women’s game (size 6). Except for the size of the ball, there were no differences in the rules based on age or sex. The winning team and losing team were analyzed separately; 2110 team performances were analyzed in total. The games’ box scores were collected from FIBA’s official website. FIBA’s official box scores have been repeatedly confirmed to be reliable [8,9,10,11,12] and used as a data source in published articles [6,13,14,15,16,17,18].

The numbers of two- and three-point shot attempts in each game were normalized to 100 possessions [19]. The number of possessions in each game was calculated as the average of the possessions of the two teams that played against each other, which were calculated using the following equation [20]:

Possessions = FGA − ORB + TO + 0.4 × FTA,(1)

where FGA represents field goal attempts, ORB represents offensive rebounds, TO represents turnovers, and FTA represents free throw attempts. The success rates of two- and three-point shots were calculated by dividing the total number of respective successful shots by the total number of respective attempted shots.

### 2.2. Statistical Analysis

Differences in mean values of two- and three-point shot attempts were analyzed by a two-way (category × year) between-subjects analysis of variance (ANOVA) followed by Ryan’s post hoc test using ANOVA4 on the Web [21]. Eta squared (ŋ^2^) was calculated as a measure of effect size in ANOVA (ŋ^2^ = 0.01, small effect; ŋ^2^ = 0.06, medium effect; ŋ^2^ = 0.14, large effect) [22]. Differences in the success rates of two- and three-point shots were analyzed by Fisher’s exact test with Hochberg’s adjustment using the R function “fisher.multcomp” in the package “RVAideMemoire”. The version of R used was R 4.0.5 for Windows [23]. The level of significance was set at *p* < 0.05.

## 3. Results

### 3.1. Number of Two- and Three-Point Shot Attempts Per 100 Possessions

**Figure 1:**
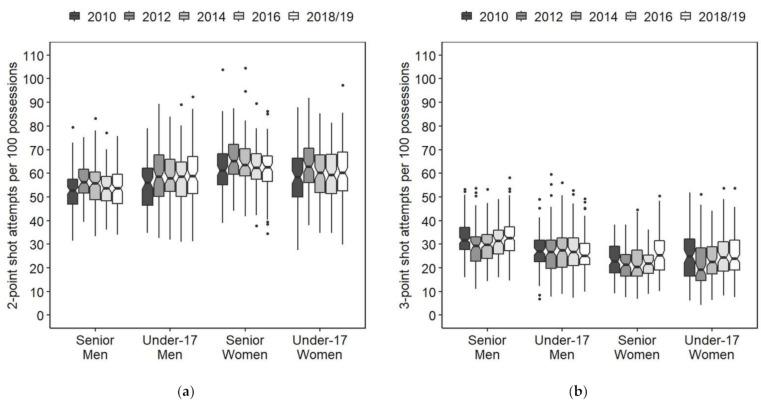
Boxplots of (**a**) two-point and (**b**) three-point shot attempts per 100 possessions for each category and year. The dots beyond the whiskers show outliers outside the 1.5 interquartile range.

**Figure 2:**
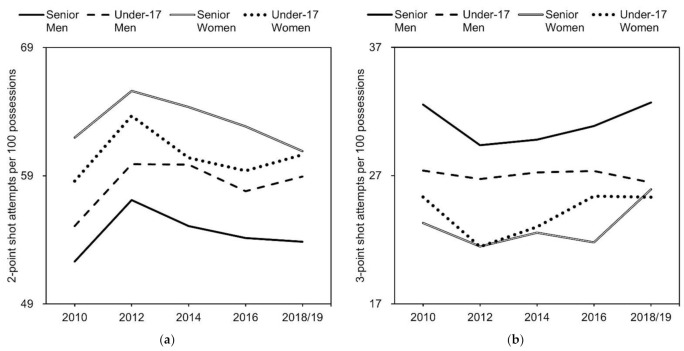
Interaction plots of (**a**) two-point and (**b**) three-point shot attempts per 100 possessions.

The numbers for two- and three-point shot attempts per 100 possessions are presented as box plots (Figure 1), interaction plots (Figure 2), and means and SDs (Table 2 and Table 3).

There was no significant category × year interaction for two-point shot attempts (*F* = 0.791, ŋ^2^ = 0.004, *p* = 0.66). However, there were significant main effects of category (*F* = 62.37, ŋ^2^ = 0.080, *p* < 0.001) and year (*F* = 10.98, ŋ^2^ = 0.019, *p* < 0.001) for two-point shot attempts. The effect sizes of the category and year were medium and small, respectively. Post hoc comparisons for the main effect of the category showed a significant difference in the number of two-point shot attempts between categories, with senior women, under-17 women, under-17 men, and senior men having the highest number in that order. Post hoc comparisons for the main effect of the year revealed that the number of two-point shot attempts increased significantly in 2012 and, in 2016, returned to a level not significantly different from that in 2010.

Although the effect size was small, there was a significant category × year interaction (*F* = 1.768, ŋ^2^ = 0.009, *p* < 0.05) for three-point shot attempts. The number of three-point shot attempts decreased significantly in 2012 and returned to a level not significantly different from 2010 by 2016 in senior men and under-17 women. Although the decrease observed in 2012 was not significant in senior women, the number of three-point shot attempts in 2018, which was not significantly different from the number in 2010, was significantly greater than the number in 2012. There was no significant change in the number of three-point shot attempts in under-17 men.

### 3.2. Success Rates of Two- and Three-Point Shots

Table 4 shows the success rate of two-point shots. The success rate of two-point shots was significantly higher in senior games than in under-17 games in 2014, 2016, and 2018/19 for men, and in 2012, 2014, 2016, and 2018 for women. There was no significant change over time, except for significant differences between 2010 and the other years in under-17 women’s games.

Table 5 shows the success rate of three-point shots. The success rate of three-point shots was significantly higher in senior games than in under-17 games in all the studied years for men, and in 2014, 2016, and 2018 for women. There was no significant change over time in the success rate of three-point shots.

## 4. Discussion

This study analyzed shot distribution and accuracy in senior and under-17 international basketball tournaments for men and women held between 2010 and 2019. The tournaments held in 2010 were played under the old three-point line (6.25 m); the rest of the tournaments were played under the new extended three-point line (6.75 m). The results showed that the number of two-point shot attempts increased in 2012, independent of categories, and the number of three-point shot attempts decreased in 2012, except for in under-17 men’s games. Changing the distance of the three-point line from the basket has been shown to alter the distribution of two- and three-point shot attempts in professional leagues [2,3]; when the distance of the three-point line is extended, the number of two-point shot attempts increases while the number of three-point shot attempts decreases, and vice versa. Although games may be played differently to some extent in international tournaments compared to professional leagues, the change in the distance of the three-point line is likely to have a similar effect on the shot distribution. However, there was an exception: the under-17 men’s tournaments showed no change in the number of three-point shot attempts. The fact that two-point shot attempts increased and three-point shot attempts did not change means that the number of free throws and/or turnovers decreased. While this result is interesting, it is unclear why different trends were seen in under-17 men.

The number of two-point shot attempts, which increased in 2012, has since begun to decline, and the number of three-point shot attempts, which declined in 2012, has since begun to increase. This period coincides with the time when the percentage of three-point shot attempts skyrocketed in the NBA. It has been argued that the increase in the percentage of three-point shot attempts in the NBA is due to the fact that more teams are focusing on efficiency based on sports analytics [4,5]. However, there were no significant differences in the number of two- and three-point shot attempts between the 2010 and the 2018/19 international tournaments. It is likely that international tournaments in the 2010s experienced a recovery from the temporary effects of the extension of the three-point line but did not experience the dramatic change in shot selection behavior seen in the NBA. However, it should be noted that three-point shot attempts per possession in the NBA in the early 2010s were less than those in international tournaments. Mandić et al. [24] compared NBA and EuroLeague data from 2000 to 2017. It can be seen that international tournaments show a trend more similar to that of the EuroLeague than that of the NBA.

Differences among categories were larger than those among years. The results of ANOVA supported this finding: the effect size of the category was larger than that of the year. The men’s tournaments showed that the number of three-point shot attempts was significantly higher in senior games than in under-17 games, except for in the 2012 tournaments. For women, on the other hand, the significant age difference in the three-point shot attempts was only found in the 2016 tournaments, in which the number of three-point shot attempts was significantly higher in under-17 games than in senior games. These results suggest that women have more similarities between senior and under-17 games than men in terms of shot distribution. Shot distribution is influenced not only by an individual player’s decisions but also by team tactics. For example, along with the increase in three-point shot attempts, the number of post-ups in the NBA has been declining since the mid-2010s [25]. Therefore, in terms of tactical adaptation, girls may have a smoother transition to senior national teams than boys. However, it is not always appropriate to have youth players perform the same things as senior players. Youth players, who are still developing physically and technically, need developmentally appropriate training [26,27]. The success rate of three-point shots was lower in under-17 games than in senior games. Previous studies have reported that three-point shot accuracy correlates with upper limb strength [28] and power [29]. It has also been reported that youth players shoot with a lower shoulder angle than senior players [30,31]. It is speculated that the lower shoulder angle compensates for a lack of muscle strength, and the compensatory movement might have detrimental effects on shooting technique development. Long-term development requires consideration of a variety of factors, not just tactical adaptation.

A limitation of this study is that since the data analyzed in this study were collected from box scores, precise shot locations in each two- and three-point area were not clear. Goldsberry [32] analyzed shot location data of NBA players and showed that the decline in two-point shot attempts in the NBA in the 2010s was primarily due to a decline in mid-range shot attempts. Although there were no significant differences in the number of two- and three-point shot attempts between the 2010 and the 2018/19 international tournaments, shot locations in each two- and three-point area might differ due to tactical evolution.

## 5. Conclusions

Overall, the extension of the three-point line had a similar effect on the shot distribution in international basketball games as it did in professional league games: two-point shot attempts increased, and three-point shot attempts decreased. The exception was the under-17 men’s tournament, where there was no change in three-point shot attempts. The altered shot distribution has recovered within a decade, but international tournaments did not experience the dramatic change in shot selection behavior seen in the 2010s NBA. The effect of the extension of the three-point line on shot accuracy was limited. Differences in shot accuracy were prominent between age categories: the accuracy was lower in under-17 games than in senior games for both sexes. Although under-17 men attempted fewer three-point shots than senior men, under-17 women attempted a similar number of or more three-point shots than senior women. These findings contribute to a better understanding of age and sex differences in recent trends in international basketball and provide a basis for an effective long-term development program. In addition, for basketball to remain an attractive sport in the future, it is important to learn from its history. In this sense, the results of this study will be valuable to all those involved in basketball.

## Figures and Tables

**Table 1:** Tournaments analyzed in this study.

Category	Year	Tournaments	Number of Games
Senior Men	2010	FIBA World Championship	80
	2012	Olympic tournament	38
	2014	FIBA Basketball World Cup	76
	2016	Olympic tournament	38
	2019	FIBA Basketball World Cup	92
Under-17 Men	2010	FIBA Under-17 World Championship	46
	2012	FIBA Under-17 World Championship	46
	2014	FIBA Under-17 World Championship	56
	2016	FIBA Under-17 World Championship	56
	2018	FIBA Under-17 Basketball World Cup	56
Senior Women	2010	FIBA World Championship for Women	62
	2012	Olympic tournament	38
	2014	FIBA World Championship for Women	40
	2016	Olympic tournament	38
	2018	FIBA Women’s Basketball World Cup	40
Under-17 Women	2010	FIBA Under-17 World Championship for Women	46
	2012	FIBA Under-17 World Championship for Women	46
	2014	FIBA Under-17 World Championship for Women	56
	2016	FIBA Under-17 World Championship for Women	49
	2018	FIBA Under-17 Women’s Basketball World Cup	56

**Table 2:** Means and SDs of two-point shot attempts per 100 possessions.

Category	2010 ^A^	2012 ^B^	2014 ^BC^	2016 ^AC^	2018/19 ^AC^
Senior Men ^a^	52.3 ± 8.0	57.1 ± 7.9	55.1 ± 9.5	54.2 ± 7.8	53.8 ± 8.3
Under-17 Men ^b^	55.1 ± 10.1	59.9 ± 12.1	59.9 ± 11.1	57.8 ± 11.5	58.9 ± 11.2
Senior Women ^c^	62.0 ± 10.8	65.6 ± 9.5	64.4 ± 10.7	62.9 ± 9.5	60.9 ± 10.1
Under-17 Women ^d^	58.6 ± 12.4	63.7 ± 11.1	60.4 ± 10.7	59.4 ± 10.9	60.6 ± 12.5

Superscript lowercase letters show the results of post hoc comparisons for the main effect of the category. Superscript capital letters show the results of post hoc comparisons for the main effect of the year. Values not sharing the same letter are significantly different from one another (*p* < 0.05).

**Table 3:** Means and SDs of three-point shot attempts per 100 possessions.

Category	2010	2012	2014	2016	2018/19
Senior Men	32.5 ± 7.3 ^a,A^	29.4 ± 9.0 ^a,B^	29.8 ± 7.5 ^a,B^	30.9 ± 7.6 ^a,AB^	32.7 ± 8.0 ^a,A^
Under-17 Men	27.4 ± 8.0 ^b,A^	26.7 ± 9.8 ^a^^,A^	27.3 ± 9.4 ^b,A^	27.4 ± 8.3 ^b,A^	26.5 ± 7.7 ^b,A^
Senior Women	23.3 ± 7.0 ^c,AB^	21.5 ± 6.3 ^b,A^	22.6 ± 8.4 ^c,AB^	21.8 ± 5.5 ^c,A^	25.9 ± 8.9 ^b,B^
Under-17 Women	25.3 ± 10.6 ^bc,A^	21.5 ± 9.7 ^b,B^	23.0 ± 8.4 ^c,AB^	25.4 ± 9.2 ^b,A^	25.3 ± 8.7 ^b,A^

Superscript lowercase letters show the results of post hoc comparisons between the categories within the same year. Superscript capital letters show the results of post hoc comparisons between the years within the same categories. Values not sharing the same letter are significantly different from one another (*p* < 0.05).

**Table 4:** Success rate of two-point shots (%).

Category	2010	2012	2014	2016	2018/19
Senior Men	50.6 ^a,A^	49.8 ^a,A^	50.8 ^a,A^	52.2 ^a,A^	50.6 ^a,A^
Under-17 Men	48.5 ^a,A^	46.3 ^ab,A^	45.2 ^b,A^	45.0 ^b,A^	45.9 ^b,A^
Senior Women	44.7 ^b,A^	43.2 ^b,A^	43.3 ^b,A^	46.4 ^b,A^	43.8 ^b,A^
Under-17 Women	43.7 ^b,A^	38.3 ^c,B^	37.3 ^c,B^	36.8 ^c,B^	39.8 ^c,B^

Superscript lowercase letters show the results of multiple comparisons between the categories within the same year. Superscript capital letters show the results of multiple comparisons between the years within the same categories. Values not sharing the same letter are significantly different from one another (*p* < 0.05).

**Table 5:** Success rate of three-point shots (%).

Category	2010	2012	2014	2016	2018/19
Senior Men	35.4 ^a,A^	35.4 ^a,A^	34.6 ^a,A^	33.7 ^a,A^	34.0 ^a,A^
Under-17 Men	30.8 ^b,A^	28.9 ^b,A^	27.7 ^bc,A^	27.1 ^b,A^	26.1 ^bc,A^
Senior Women	31.9 ^ab,A^	30.3 ^ab,A^	31.7 ^ac,A^	35.9 ^a,A^	31.0 ^ac,A^
Under-17 Women	28.6 ^b,A^	26.5 ^b,A^	24.9 ^b,A^	24.9 ^b,A^	25.3 ^b,A^

Superscript lowercase letters show the results of multiple comparisons between the categories within the same year. Superscript capital letters show the results of multiple comparisons between the years within the same categories. Values not sharing the same letter are significantly different from one another (*p* < 0.05).

## Data Availability

The data analyzed in this study were collected from the publicly accessible website of the International Basketball Federation.
